# A Human Integrin-α3 Mutation Confers Major Renal Developmental Defects

**DOI:** 10.1371/journal.pone.0090879

**Published:** 2014-03-12

**Authors:** Rachel Shukrun, Asaf Vivante, Oren Pleniceanu, Einav Vax, Yair Anikster, Benjamin Dekel, Danny Lotan

**Affiliations:** 1 Pediatric Stem Cell Research Institute, Edmond and Lili Safra Children's Hospital, Sheba Medical Center, Ramat Gan, Israel; 2 Sheba Centers for Regenerative Medicine and Cancer Research, Sheba Medical Center, Ramat Gan, Israel; 3 Department of Pediatrics and Pediatric Nephrology, Safra Children's Hospital, Sheba Medical Center, Ramat Gan, Israel; 4 Sackler School of Medicine, Tel Aviv University, Tel Aviv, Israel; University of Vienna, Max F. Perutz Laboratories, Austria

## Abstract

The development of the mammalian kidney is a highly complex process dependent upon the interplay of various cell types, secreted morphogens, and the extra-cellular matrix (ECM). Although integrins are the most important receptors for ECM proteins and are ubiquitously expressed during kidney development, mice lacking expression of integrin α3 (Itga3) do not demonstrate a reduced number of nephrons, but mostly a disorganized GBM (glomerular basement membrane) leading to proteinuria. Thus, ITGA3 is considered mostly a passive GBM stabilizer and not an active player in nephrogenesis. Recently, mutations in the human *ITGA3* were shown to cause congenital nephrotic syndrome, epidermolysis bullosa and interstitial lung disease, otherwise termed NEP syndrome (**N**ephrotic syndrome, **E**pidermolysis bullosa and **P**ulmonary disease). Herein, we performed histological and molecular analysis on the kidneys of a single patient from the initial cohort harboring an *ITGA3* mutation, to illuminate the role of *ITGA3* in human renal development. We show the patient to harbor a unique phenotype at birth, including severe unilateral renal hypodysplasia. Interrogation of global gene expression in the hypodysplastic kidney versus three controls (fetal, child and adult kidneys) revealed perturbed expression in several renal developmental pathways implicated in hypodysplasia, including the Wnt, BMP (bone morphogenetic protein) and TGF (transforming growth factor) pathways. Moreover, the affected kidney showed upregulation of early embryonic genes (e.g. *OCT4* and *PAX8*) concomitant with downregulated kidney differentiation markers, implying a defect in proper renal differentiation. In conclusion, we show for the first time that ITGA3 is not merely a passive anchor for renal ECM proteins, as predicted by mouse models. Instead, our results may suggest it plays a central role in the interplay of cells, morphogens and ECM, required for proper nephrogenesis, thus adding *ITGA3* to the list of CAKUT (congenital anomalies of the kidney and urinary tract)-causing genes.

## Introduction

The formation of the metanephric kidney occurs via the concerted actions of several important factors. Two precursor tissues, the metanephric mesenchyme (MM) and ureteric bud (UB) interact with each other to allow the generation of approximately 900,000 to 1 million nephrons [1], [2]. In addition, this process of nephrogenesis involves multiple secreted factors, including members of the WNT, BMP (bone morphogenetic protein) and TGF (transforming growth factor) protein families [3]. Finally, extra-cellular matrix (ECM) proteins participate in regulation of renal development, by generation and modulation of various cellular activities [4].

The integrin family of receptors forms a diverse group of molecules, which constitute the main family of receptors for ECM proteins [5]. The kidney has some of the most complex ECM, composed mainly of type IV collagen, laminins, nidogen, and proteoglycans [5]. Thus, it is not surprising that integrins are ubiquitously expressed in the kidney, with integrin α3β1 constitutes the most abundant renal integrin [5], [6]. Early studies in rodents [7] demonstrated that integrin α3β1 is crucial for the podocyte-GBM (glomerular basement membrane) interaction and thus essential for maintaining the glomerular filtration barrier. Both *Itga3*-null mice and mice lacking *Itga3* expression specifically in podocytes develop massive proteinuria secondary to severe podocytopathy and disorganization of the GBM [7], [8]. These findings are consistent with the high levels of Itga3 expression in immature podocytes, endothelial, and mesangial cells during kidney development [6].Although *Itga3*-null mice show decreased branching of the medullary collecting ducts, the number of nephrons is normal [7], suggesting that aside from its role in GBM formation and stabilization, *ITGA3* has a rather limited role in nephrogenesis and thus is not considered a CAKUT (congenital anomalies of the kidney and urinary tract)-causing gene. This assertion was confirmed when mice lacking Itga3 specifically in UB cells demonstrated a surprisingly subtle phenotype, showing decreased papillary outgrowth [9]. This allegedly minimal role of *ITGA3* in nephron development was unexpected for two main reasons. First, Itga3 is expressed in several key regions of the developing kidney (e.g. undifferentiated MM, primary vesicles, S-shaped bodies and developing tubules) [6]. Second, as previously mentioned, kidney development is highly dependent upon reciprocal interactions between the UB and MM, thus requiring complex cell–ECM interactions [10].

Recently, however, *ITGA3* homozygous mutations were reported [11] in three patients with a multi-organ disorder comprised of congenital nephrotic syndrome, epidermolysis bullosa and interstitial lung disease, or NEP syndrome (**N**ephrotic syndrome, **E**pidermolysis bullosa and **P**ulmonary disease). In contrast to classical congenital nephrotic syndrome patients, in which kidney ultrasound reveals enlarged kidneys [12], in two out of the three patients, postnatal ultrasound examination demonstrated unilateral or bilateral renal hypodysplasia, suggestive of a concomitant congenital anomaly of kidney development [11]. More recently, a similar phenotype, consisting of interstitial lung disease and nephrotic syndrome was reported in child carrying a missense *ITGA3* mutation that led to gain of glycosylation in the α3 subunit [13].

The availability of human kidney tissue from a patient harboring an *ITGA3* mutation afforded the opportunity to characterize the renal developmental defect involved in ITGA3 deficiency at both the histological and genetic levels. These findings allowed us, to delineate a possible role for *ITGA3* in human nephrogenesis and study the developmental pathways affected by its dysregulation. We investigated ITGA3 protein localization in developing human kidneys. In addition we used renal tissue originally obtained from a patient with an *ITGA3* mutation and assessed its gene expression profile as compared to controls. The strikingly severe and previously undescribed hypodysplastic-nephrotic phenotype (usually presenting as two separate disease entities) and significantly deranged global gene expression profile suggest that ITGA3 is an essential regulator of nephrogenesis and not merely a passive component of the GBM. These findings illustrate the complex reciprocal interactions that take place during human kidney development in the face of a single gene mutation

## Methods

### Ethics Statement

This study was conducted according to the principles expressed in the Declaration of Helsinki. The study was approved by the Institutional Review Board of Sheba Medical Center (SMC- 9367-12) and Asaf Harofeh Medical Center (71/31) hospitals. All patients provided written informed consent for the collection of samples and subsequent analysis. The individual in this manuscript has given written informed consent (as outlined in PLOS consent form) to publish these case details.

### Patients' DNA and Kidney Tissue

The current study involved the usage of blood and tissue samples obtained from a previously reported patient [11] who died due to multiple organ failure at the age of 2 months, secondary to NEP-Syndrome complications. Post mortem genetic diagnosis revealed Integrin α3 mutations (c.1538-1G→A).

### Human Fetal and Adult Kidney Tissue

Normal human 16 week gestation kidney was obtained following curettage of elective abortions. Normal human adult kidney samples were retrieved from borders of renal cell carcinoma tumors from patients who underwent partial nephrectomy. Fetal and adult kidney tissues were handled within 1 h following the curettage or nephrectomy procedures, respectively. All studies were approved by the local ethical committee and informed consent was provided by the patients involved in this research according to the Declaration of Helsinki. Collected tissues were washed with cold Hank's Balanced Salt Solution (HBSS) (Invitrogen, Carlsbad, Calif., USA) and cut into 0.5 cm cubes by sterile surgical scalpels. The pieces were then used for total RNA extraction with TRIzol (Life Technologies, Invitrogen, Carlsbad, Calif., USA)

### DHPLC - Denaturing High-Performance Liquid Chromatography

Scanning for DNA mutations and variants using DHPLC involves subjecting polymerase chain reaction (PCR) products to chromatography using an ion-pair reversed-phase cartridge (PCR primers are available upon request). PCR products are denaturized and allowed to re-anneal. Under conditions of partial denaturation with a linear acetonitrile gradient, heteroduplexes from PCR samples with internal sequence variation display reduced column retention time relative to their homoduplex counterparts. The elution profile for a heterozygous sample is typically quite distinct from that of either homozygous sequence, making identification of heterozygous mutations relatively straightforward.

### Chip Array

The chip array data is deposited in publicly library (GEO), accession number GSE54227. All chip array experiments were performed using Affymetrix HU GENE1.0st oligonucleotide arrays (www.affymetrix.com/support/technical/datasheets/gene_1_0_st_datasheet.pdf). Total RNA was extracted from each of the four kidney samples: the index patient's kidney, a normal age-matched kidney, a normal fetal kidney and a normal adult kidney. These RNA samples were used to prepare biotinylated target DNA, according to manufacturer's recommendations. The target complementary DNA (cDNA) generated from each sample was processed as per manufacturer's recommendation using an Affymetrix GeneChip Instrument System (www.affymetrix.com/support/downloads/manuals/wt_sensetarget_label_manual.pdf). RNA quality and amount were confirmed using an agarose gel or by Bioanalyzer (Agilent). After scanning, array images were assessed by eye to confirm scanner alignment and the absence of significant bubbles or scratches on the chip surface. The signals derived from the array were assessed using various quality assessment metrics. Gene-level Robust Multi-array Average (RMA) sketch algorithm (Affymetrix Expression Console and Partek Genomics Suite 6.2) was used for crude data generation. Significantly changed genes were filtered as changed by at least twofold (p-value: 0.05). Genes were filtered and analyzed using unsupervised hierarchical cluster analysis and supervised hierarchical cluster analysis (Partek Genomics Suite and Spotfire DecisionSite for Functional Genomics) to get a first assessment of the data. Predictions of functionality were performed by using functional analysis and overrepresentation calculations based on gene ontology (GO) and publication data: DAVID (Database for Annotation, Visualization and Integrated Discovery, http://apps1.niaid.nih.gov/David/upload.asp), Ingenuity, Database for Annotation (GO), Visualization, and Integrated Discovery. Overrepresentation calculations were done using Ease (DAVID).

### Hematoxylin and eosin (H&E) staining

H&E staining of paraffin-embedded kidney tissues of the index patient: 5 µm sections of paraffin-embedded tissues were mounted on super frost/plus glass and incubated at 60°C for 40 minutes. After deparaffinization, slides were incubated in Mayer's Hematoxylin solution (Sigma-Aldrich) and incubated with 1% Hydrochloric acid in 70% ethanol for 1 minute. Slides were then incubated for 10 seconds in Eosin (Sigma-Aldrich). Images were produced using Olympus BX51TF.

### Immunohistochemical staining

Sections, 5-µm thick, were cut from human adult kidney, human fetal kidney and both kidneys from the index patient for immunohistochemistry. Immunostainings were performed as previously described [14]. Briefly, the sections were processed within 1 week to avoid oxidation of antigens. Before immunostaining, sections were treated with 10 mM citrate buffer, pH 6.0 for 10 min at 97°C in a microwave oven for antigen retrieval, followed by 3% H_2_O_2_ for 10 min. The slides were subsequently stained using the labeled strepavidin-biotin (LAB-SA) method using a Histostain plus kit (Zymed, San Francisco, CA, USA). Anti human integrin α3β1 antibody (Mouse monoclonal P1B5, Abcam), at a dilution of 1∶100, were used. Controls were prepared by omitting the primary antibodies or by substituting goat IgG isotype for the primary antibodies. The immunoreaction was visualized by an HRP (horseradish peroxidase)-based chromogen/substrate system (liquid DAB [Diaminobenzidine] substrate kit – Zymed, San Francisco, CA, USA).

## Results

### NEP syndrome comprises a renal hypodysplasia phenotype

**Figure 1:**
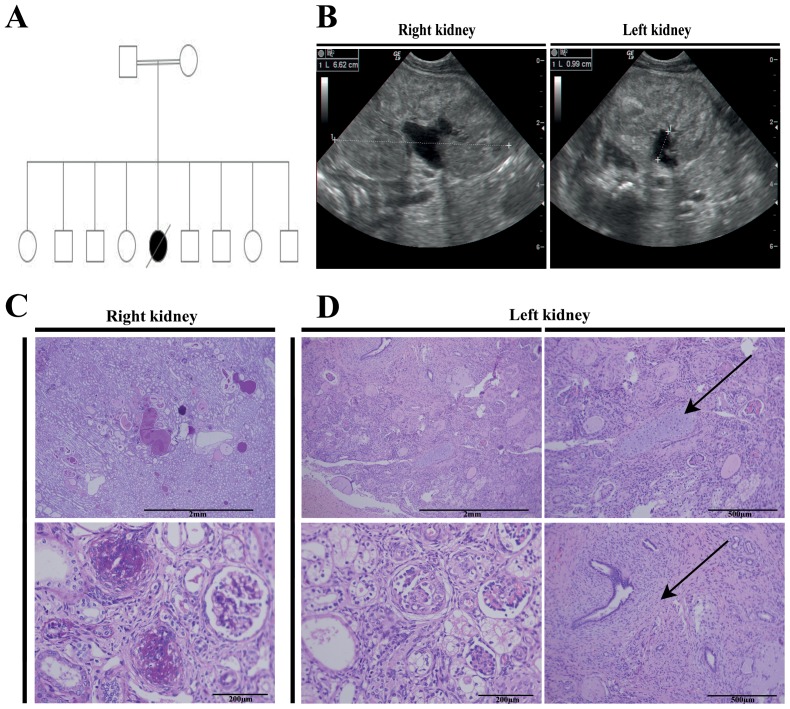
Clinical and histological features of the index patient. (A) A pedigree presenting the patient, born to healthy consanguineous parents, as the only affected child among nine siblings. (B) Renal ultrasound examination, demonstrating a small hyper-echogenic left kidney and an enlarged right kidney. (C) H&E staining of the patient's right kidney demonstrating a typical nephrotic syndrome phenotype including global sclerosis and mesangial proliferation. (D) The patient's left kidney presents histology consistent with renal hypodysplasia including the presence of cartilage, stroma and renal lesions of nephrotic syndrome similar to those observed in the right kidney.

The index patient is the only affected child among nine siblings, demonstrating an autosomal recessive inheritance pattern (Fig. 1A). In addition to NEP syndrome, the patient had a small hyper-echogenic left kidney demonstrated by renal ultrasound examination, suggestive of severe renal hypodysplasia (Fig. 1B). Because renal hypodysplasia is not included in the phenotypic spectrum of NEP syndrome [11], further investigation was performed on tissue samples obtained during post mortem analysis. H&E and silver staining of the patient's right enlarged kidney demonstrated histological features consistent with congenital nephrotic syndrome (Fig. 1C, Figure S1). The left kidney disclosed histology consistent with renal hypodysplasia including the presence of stroma, smooth muscle and patches of cartilage (Fig. 1D). Interestingly, within the left hypodysplastic kidney, renal lesions of nephrotic syndrome similar to those observed in the right kidney were detected (Fig. 1D). Having observed this renal developmental phenotype we screened nine candidate kidney developmental genes known to be associated with renal hypodysplasia. Included were: *PAX2, HNF1B, EYA1, SIX1, SIX2, SALL1, GDNF, WNT4* and *WT1* [15]. The latter analysis was negative for disease causing mutations. Taken together, these clinical, histological and molecular data indicate that the *ITGA3* mutation results in an early renal developmental defect beyond the previously described glomerular defect.

### ITGA3 expression in the developing human kidney

**Figure 2:**
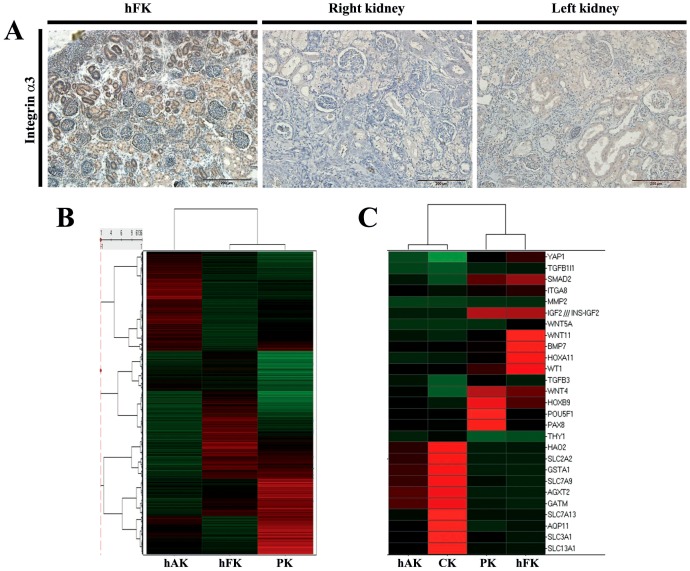
Immuno-localization and interrogation of global gene expression of the patient's kidney. (A) Immunohistochemical staining for integrin α3 reveals a widespread expression pattern in the developing human fetal kidney (hFK), with localization to early duct precursors, ureteric buds and their differentiated derivatives and basement membrane of assembled fetal glomeruli. Integrin α3 expression was absent in the patient's kidneys. (B) Heat-map comparison of gene expression profile between the patient's kidney (PK) and an age matched control (CK) kidney. Unsupervised hierarchical clustering demonstrates that the PK is more similar genetically to human fetal kidney (hFK) than to the human adult kidney counterpart (hAK). (C) Microarray expression analysis of selected genes demonstrated altered expression in the PK of genes crucial for normal nephron formation, including the Wnt and TGFβ signaling pathways, early developmental genes and renal differentiation genes.

Having shown that the *ITGA3* mutation is associated with severe defects in nephrogenesis, we next attempted to better characterize its role in renal development. To this end, we interrogated the expression domain of ITGA3 in human kidneys. As expected, immunohistochemical staining of ITGA3 in the patient's kidney showed lack of protein expression (Fig. 2A). Analysis of mid-gestation human fetal kidney (hFK) sections, that contain all phases of nephrogenesis, revealed a widespread expression pattern localized to two main compartments; 1. within early duct precursors, ureteric buds and their differentiated derivatives; 2. within the basement membrane of assembled fetal glomeruli (Fig. 2A, Figure S2). These results correlate with previously published papers demonstrating high expression in similar kidney compartments in rodent models [7], [16], [17], [18]. However, while in mice ITGA3 deficiency affects mostly the glomeruli, in humans the effect is more severe with wide-spread hypodysplasia. Taken together, these results suggest that *ITGA3* might play a more significant role in human nephrogenesis than first predicted by murine models.

### Expression profile of the patient's kidney reveals insights into ITGA3 contribution to renal development

We next sought to decipher the molecular link between the *ITGA3* mutation and the patient's renal phenotype showing renal hypodysplasia. To this end, we characterized the global gene expression profile of the patient's kidney by performing microarray analysis. Remarkably, a comparison between the patient's kidney (PK) and a normal age-matched control kidney (CK) uncovered 4,042 differentially expressed genes; indicating massively dysregulated gene expression in the *ITGA3*-mutated kidneys. Hierarchical clustering of these gene profiles demonstrated that the patient's kidney is genetically closer to hFK than to the human adult kidney (hAK) counterpart (Fig. 2B), suggestive of developmental arrest. Analysis of differentially expressed genes using Ingenuity© showed that 526 of these 4,042 genes are involved in cellular development. Importantly, gene expression differences between the samples implicated several pathologic processes important in the development of human renal dysplasia. These include altered expression of genes necessary for normal nephron formation, such as *BMP7* and *WT1*; up regulation of key regulators of UB branching, such as *HOXA11* and *ITGA8*; increased expression of genes involved in the Wnt pathway, such as *WNT5A*, *WNT4* and *WNT11*, and genes involved in the TGFβ signaling pathway, such as *TGFB1I1*, *TGFB3*, *SMAD2* and *MMP2* (Fig. 2C). These changes imply great resemblance to renal dysplasia phenotype [19] with marked differences between the patient and the age matched control. In addition, among the most increased transcripts in the PK relative to the CK were early developmental genes such as *OCT4* (*POU5F1*), *HOXB9*, *PAX8*, *YAP1* and *IGF2*. Genes identifying differentiated proximal tubules in the kidney such as *GATM*, *AGXT2*, *GSTA1* (glycine, alanine-glyoxylate and glutathione aminotransferases respectively), *SLC3A1*, *SLC13A1*, *SLC7A9*, *SLC2A2* (members of the solute carrier family) and *AQP11* (aquaporin 11) were among the most down regulated transcripts in the PK (Fig. 2C). Taken together, these findings indicate that ITGA3 deficiency leads to an early developmental defect due to dysregulation of several key pathways in nephrogenesis, resulting in renal hypodysplasia/CAKUT phenotype.

## Discussion

In this study, we have highlighted and investigated *ITGA3* disease causing mutations in humans and demonstrated that such mutations can lead to a renal phenotype across the spectrum of CAKUT. Among two of the three patients initially reported to harbor *ITGA3* mutations, postnatal ultrasound examination demonstrated unilateral or bilateral renal hypodysplasia, suggestive of a concomitant congenital anomaly of kidney development [11]. In addition, in a recent subsequent report of a patient with a novel missense mutation of *ITGA3*, which lead to fatal interstitial lung disease and congenital nephrotic syndrome, renal phenotype per ultrasound revealed unilateral left kidney hypodysplasia with hydronephrosis, similar to the patient in the current report [13]. Moreover, the renal hypodysplasia phenotype presented here was confirmed by histopathology and no other known renal hypodysplasia disease or CAKUT causing mutations were found.

An early renal developmental role of *Itga3* was somewhat underestimated in mouse models which link the major renal phenotype to the role of Itga3 in the establishment and maintenance of GBM integrity [7]. It cannot be excluded that the massive nephrosis, a consequence of the mal-developed GBM, can induce interstitial fibrosis in the kidneys and contribute to a dysplastic phenotype. However, the unique overlapping hypodysplastic/nephrotic phenotype in which kidneys can presumably come from 2 different patients has not been described in other congenital nephrotic syndromes in which the defective protein is localized to glomeruli and not to early kidney precursors (e.g. nephrin, podocin) [20]. In addition, our results are consistent with the widespread distribution of integrin α3β1 in the developing kidney. Moreover, mutations in the laminin α5 chain (a subunit of laminin-10/11, both ligands of integrin α3β1) have been shown to cause a similar combined phenotype of renal agenesis and defective glomerulogenesis [21]. In conclusion, although validation of the findings in additional patients is required, our results suggest a more central role for *ITGA3* in human renal development than previously described, and provide an insight into the developmental pathways that are affected by its absence. From a practical point of view, *ITGA3* may be not only exclusively included in congenital nephrotic syndrome genes screen but also in a large survey of CAKUT-causing mutations.

## Supporting Information

Figure S1 **Silver staining of the patient's right kidney.** (A) Silver staining of the patient's right kidney demonstrating thickening of the glomerular basement membrane and mesangial expansion. These results are compatible with a nephrotic syndrome phenotype.
(TIF)

Figure S2 **Integrin α3 expression in the collecting system of human fetal kidney.** (A) Immunohistochemical staining for integrin α3 reveals a widespread expression pattern in the papilla of developing human fetal kidney, with localization to collecting ducts and their derivatives.
(TIF)
