# Supplementary figures and images for: A Human Integrin-α3 Mutation Confers Major Renal Developmental Defects
Source: PLoS One. 2014 Mar 12;9(3):e90879. doi: 10.1371/journal.pone.0090879 (PMC3951280; doi:10.1371/journal.pone.0090879)

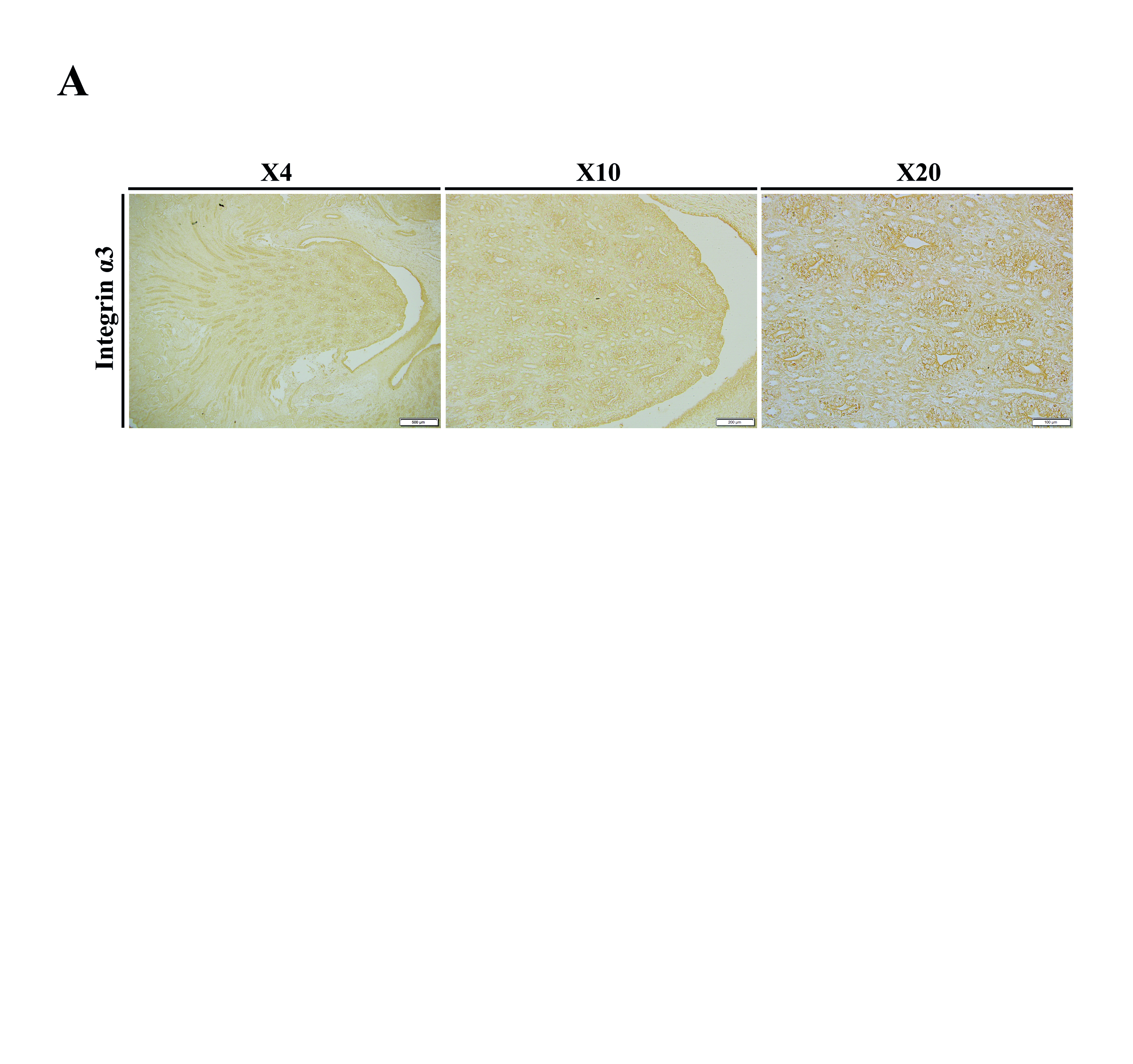

Supplement: Figure S1 — Silver staining of the patient's right kidney. (A) Silver staining of the patient's right kidney demonstrating thickening of the glomerular basement membrane and mesangial expansion. These results are compatible with a nephrotic syndrome phenotype. (TIF) [file pone.0090879.s001.tif]

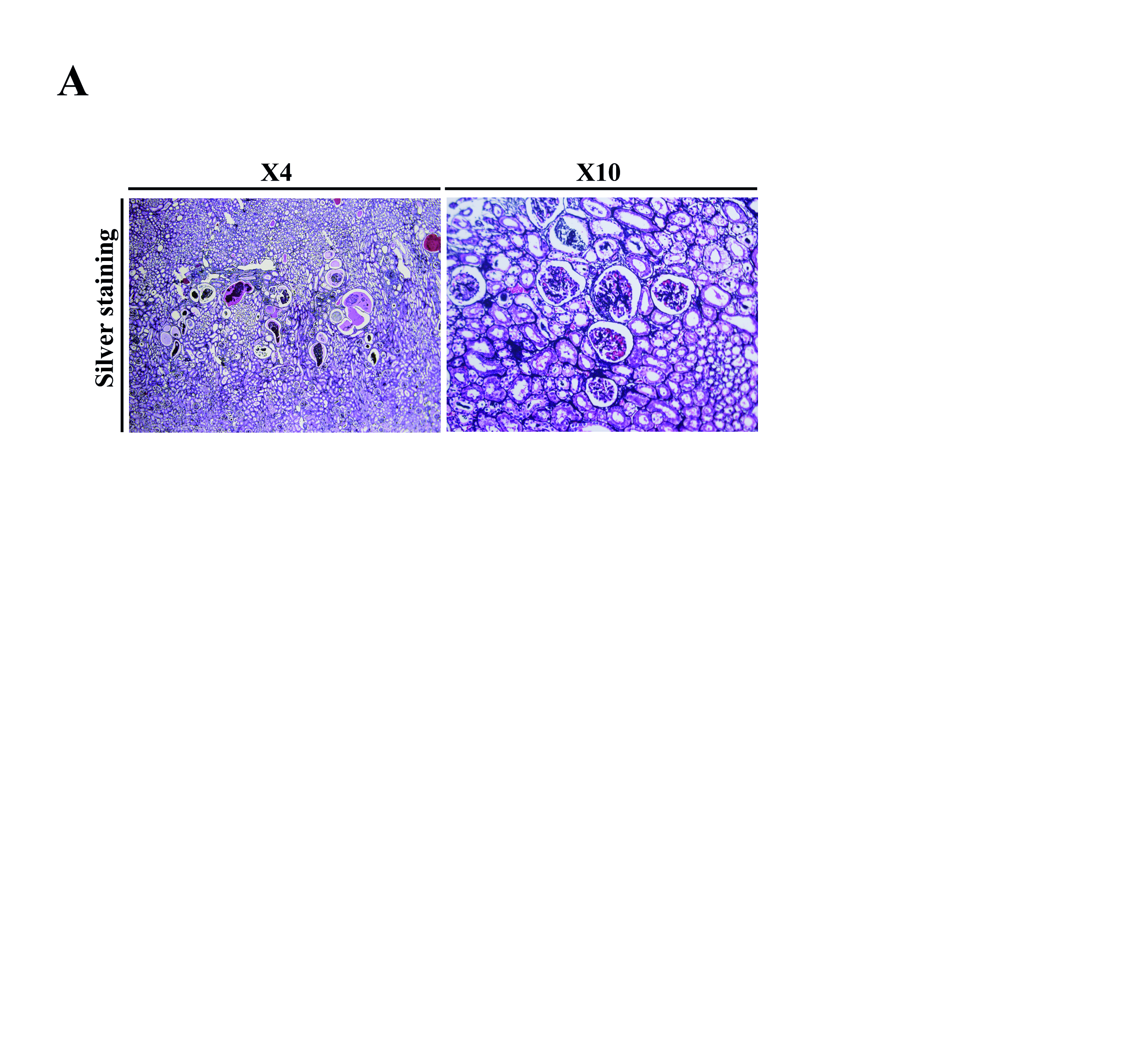

Supplement: Figure S2 — Integrin α3 expression in the collecting system of human fetal kidney. (A) Immunohistochemical staining for integrin α3 reveals a widespread expression pattern in the papilla of developing human fetal kidney, with localization to collecting ducts and their derivatives. (TIF) [file pone.0090879.s002.tif]
